# A new method for segmentation and analysis of bone callus in rodent fracture models using micro‐CT

**DOI:** 10.1002/jor.25507

**Published:** 2023-01-11

**Authors:** Mark Hopkinson, Gareth Jones, Lucinda Evans, Stephanie Gohin, Ran Magnusdottir, Phil Salmon, Chantal Chenu, Richard Meeson, Behzad Javaheri, Andrew A. Pitsillides

**Affiliations:** ^1^ Comparative Biological Sciences Royal Veterinary College London UK; ^2^ Clinical Science and Services Royal Veterinary College London UK; ^3^ Bruker‐microCT Kontich Belgium; ^4^ Division of Surgery University College London London UK; ^5^ Present address: School of Mathematics, Computer Science and Engineering City University of London London UK

**Keywords:** automated segmentation, bone, callus, fracture, micro‐CT

## Abstract

Fracture burden has created a need to better understand bone repair processes under different pathophysiological states. Evaluation of structural and material properties of the mineralized callus, which is integral to restoring biomechanical stability is, therefore, vital. Microcomputed tomography (micro‐CT) can facilitate noninvasive imaging of fracture repair, however, current methods for callus segmentation are only semiautomated, restricted to defined regions, time/labor intensive, and prone to user variation. Herein, we share a new automatic method for segmenting callus in micro‐CT tomograms that will allow for objective, quantitative analysis of the bone fracture microarchitecture. Fractured and nonfractured mouse femurs were scanned and processed by both manual and automated segmentation of fracture callus from cortical bone after which microarchitectural parameters were analyzed. All segmentation and analysis steps were performed using CTAn (Bruker) with automatic segmentation performed using the software's image‐processing plugins. Results showed automatic segmentation reliably and consistently segmented callus from cortical bone, demonstrating good agreement with manual methods with low bias: tissue volume (TV): −0.320 mm^3^, bone volume (BV): 0.0358 mm^3^, and bone volume/tissue volume (BV/TV): −3.52%, and was faster and eliminated user‐bias and variation. Method scalability and translatability across rodent models were verified in scans of fractured rat femora showing good agreement with manual methods with low bias: TV: −3.654 mm^3^, BV: 0.830 mm^3^, and BV/TV: 7.81%. Together, these data validate a new automated method for segmentation of callus and cortical bone in micro‐CT tomograms that we share as a fast, reliable, and less user‐dependent tool for application to study bone callus in fracture, and potentially elsewhere.

## 1 INTRODUCTION

Formation of mineralized callus is an integral part of indirect fracture repair, providing the bone with biomechanical stability, restoring limb function, and enabling later bone remodeling. The fracture callus is therefore a highly dynamic environment with changing proportions of tissues with varying architectural and material properties, which together determine the robustness of the repair tissue and ultimately the progress of bone regeneration. An appreciation of both the structural and material properties of the callus and repaired bone by appropriate analyses is therefore vital in developing our understanding of the fracture repair processes under different pathophysiological states and experimental settings.^1^

Effective assessment of fracture repair requires evaluation of strength and stiffness of the fracture callus and repaired bone within the integrated context of whole bone structure. However, these mechanical parameters are currently difficult to measure directly in clinical settings.^2^ Current clinical fracture assessment predominantly involves use of plain film X‐ray imaging,^3^ relying on two‐dimensional (2D) images to assess 3D structures. The noninvasive, nondestructive method of computed tomography (CT) imaging can produce an array of 3D evaluations of structure and measurements of local mineralization. These can be used to indirectly estimate predicted strength and stiffness of the fracture callus and repaired bone. In a clinical research setting, quantitative computed tomography (QCT) or peripheral QCT (pQCT) have been used to assess bone mineral density (BMD) during fracture repair.^4^ These studies have established that callus size, bone mineral content (BMC), and BMD from pQCT are useful surrogate measures of compressive failure load.^5^ Micro‐computed tomography (micro‐CT) provides far greater resolution than clinical CT scanners and is ideal for studying the architecture of woven (irregular) bone in callus, particularly in the context of small animals. Several studies have successfully utilized micro‐CT to measure an array of parameters associated with the architecture and mineralization of developing callus, with Morgan et al.^2^ identifying bone volume (BV), bone volume/tissue volume (BV/TV), and tissue mineral density as the strongest predictors of fracture callus mechanical strength.^2^, ^6^, ^7^

In a research setting, micro‐CT analysis provides a wealth of data and insight into the structure and mineralization of both repairing cortical bone and callus. However, current methods for appropriate segmentation of callus from cortical bone rely on semiautomated methods applied to defined regions of interest (ROIs).^2^, ^8^, ^9^, ^10^, ^11^ Defining these regions requires users to manually specify the boundary of the callus—where it meets endosteal and periosteal surfaces of the original bone—in multiple 2D images which makes this process both time‐consuming and prone to operator error. Inappropriate segmentation results in inaccurate insights into healing progression and mechanical properties of the repairing bone. A novel automated method that addresses some of these limitations has been described.^12^ The segmentation method that this elegant study employed is, nonetheless, reliant upon multilevel global thresholding and uses a single thresholding step with a view to define callus based on mineral density alone, which has limitations. These limitations may indeed prove problematic, as the callus increases in mineral density and radio‐opacity during healing, making its discrimination from original or repaired bone highly subjective when reliant upon greyscale thresholding.

The need for quantitative evaluation of the fracture healing in an experimental setting, coupled with the lack of a ready method for selective and fast segmentation of mineralized callus, establishes a bottleneck in scope to monitor, understand and improve fracture healing processes, which may inform clinical therapies. Herein, we describe a newly developed, fully automated method to segment and separately analyse the callus and cortical bone from micro‐CT images. This method provides innovation as, unlike previous automated approaches, it is neither limited by the need for user ROI contouring nor by any exclusive reliance upon thresholding, to facilitate discrimination between callus and cortical bone.

## 2 METHODS

### 2.1 Animals and experimental protocols

All animal experiments were conducted under license from the Home Office (UK) in accordance with The Animals (Scientific Procedures) Act 1986 and were approved by the Institutional Ethics and Welfare Committee Animal Welfare Ethical Review Board at the Royal Veterinary College.

**Figure 1:**
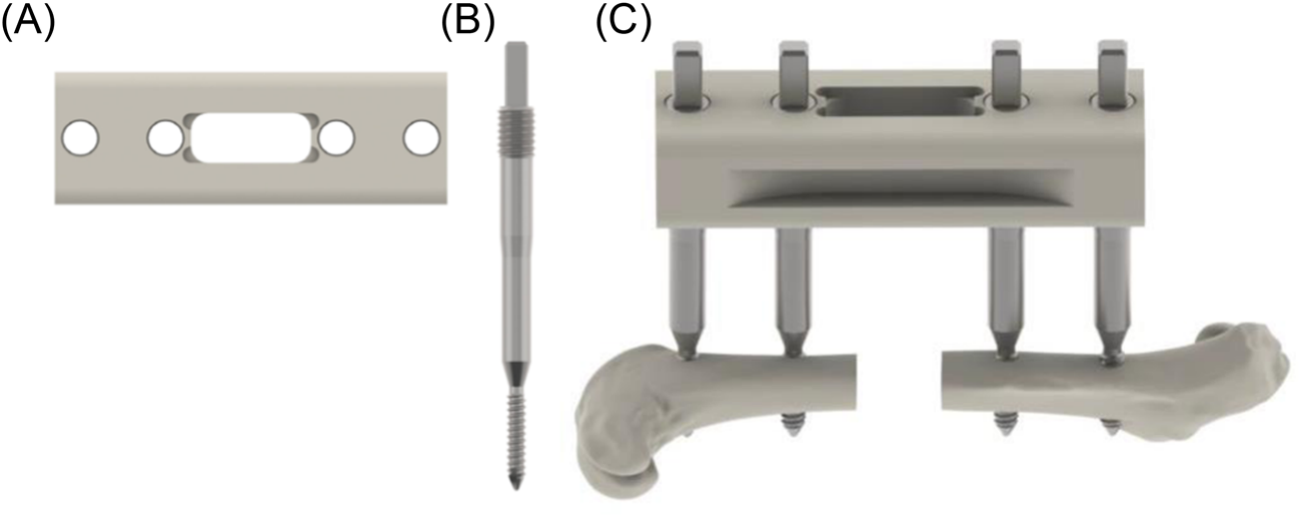
RISystem MouseExFix L external fixator system for femoral osteotomies in mice. (A) MouseExFix L external fixator—top view, (B) MouseExFix Mounting Pin, and (C) MouseExFix external fixator with pins and a large transverse osteotomy. Figure adapted from RISystem AG. [Color figure can be viewed at wileyonlinelibrary.com]

Five 11–12‐week‐old male C57BL/6 mice were anesthetized with 4% isofluorane (4 L/min oxygen) and maintained with 1.5%–2% isofluorane (2 L/min oxygen) throughout surgery. After induction of anesthesia, buprenorphine was administered subcutaneously (100 µl, 0.3 mg/ml, Vetergesic; Ceva Animal Health); the right thigh was shaved and cleaned using iodine. Femoral fractures were generated using a model devised and described by Zwingenberger et al.,^13^ in which postfracture stabilization was achieved using application of an external fixator^14^ (Figure 1). Postsurgery X‐rays were performed under anesthesia to ensure correct fixator positioning. Mice were euthanized 6 weeks postsurgery using CO_2_ and femora dissected and fixed for 48 h in 4% PFA before long‐term storage in 70% ethanol. Fixed samples had the external fixator removed and surrounding tissue around the bone removed.

Six female, 10–12‐week‐old, Wistar rat femurs were subjected to a well‐established fracture healing model using an open femoral osteotomy with a 1.5 mm fracture gap.^15^ They were anesthetized with 4% isofluorane (4 L/min oxygen) and maintained with 1.5%–2% isofluorane (2 L/min oxygen) throughout surgery. After induction of anesthesia, buprenorphine was administered subcutaneously (100 µl, 0.3 mg/ml, Vetergesic; Ceva Animal Health). Briefly, the rats had a modified Harrison type 1a external fixator applied on the craniolateral aspect of the left femora^16^ after surgical osteotomy and distraction to the required gap size. With appropriate analgesia, the rats had unrestricted exercise for 5 weeks, before being euthanized. Femora were retrieved and fixed in 10% buffered formaldehyde for 3 days before scanning.

### 2.2 Scanning

Samples were wrapped in plastic film before scanning to prevent drying and scanned using a Skyscan 1172F (Bruker). Mouse femora were scanned with X‐ray settings 50 kV and 200 µA, using an aluminum 0.5 mm filter and exposure time 960 ms using a pixel size of 5 µm. Rat femora were scanned with X‐ray settings of 60 kV and 167 μA, using a 0.5 mm aluminum filter, 1180 ms exposure time, and 4.89 μm pixel size. Projection images were reconstructed into tomograms using NRecon 1.7.3.1 (Bruker) using a reconstruction threshold with a minimum value of −1000 and maximum value of 9486 in Hounsfield units and repositioned using Dataviewer 1.5.4 (Bruker).

**Figure 2:**
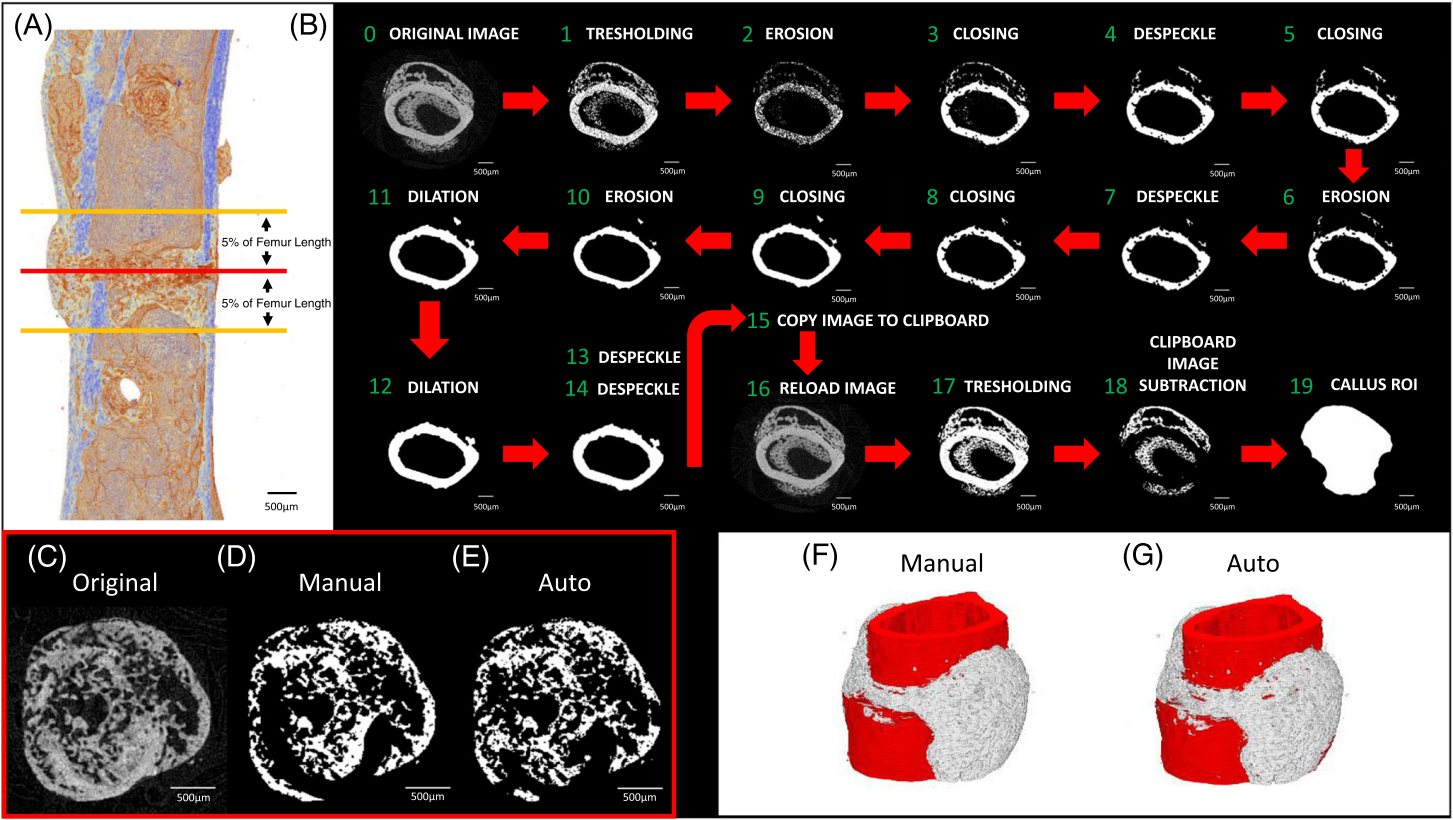
Diagrammatic representation of the various steps in the automated method. (A) Selection of the volume of interest, (B) image processing steps listed in the methods, (C) callus area where cortical bone is difficult to define, with some difference seen in (D) manual and (E) automatic segmentation of callus from cortical bone, (F) volume rendering of manual segmentation of callus in white and cortical bone in red, (G) volume rendering of automatic segmentation of callus in white and cortical bone in red. [Color figure can be viewed at wileyonlinelibrary.com]

Before any segmentations, volumes of interest (VOIs) were selected within CTAn 1.18.4 (Bruker). In mouse femora, the fracture was identified and was used as a reference point for the selection of a 10% portion of the femur length extending both proximally and distally as the VOI (Figure 2A). A volume of 10% of femur length was selected to include the entire callus region but ensuring not to include regions that contained callus formation due to the external fixator pins. As the left femur had no callus the equivalent region was selected as a VOI. In rat femora, the presence of the external fixator during scanning caused X‐ray diffraction artifacts at sites in close proximity, therefore selection of a VOI required the center of the fracture gap to be identified as a reference point for the selection of 300 slices extending both proximally and distally (total of 600 slices, 5‐μm thick slices) as the VOI. After selection, VOIs underwent segmentation, both manually and using the automated method (as described below) to separate callus from cortex (Figure 2B). This process was completed using CTAn 1.18.4 (Bruker) but the outlined steps can readily be performed in any software with segmentation capabilities (e.g., ImageJ). Volume‐rendered 3D visualizations were created using CTVox 3.3 (Figure 2C–F) (Bruker). It is important to note that the automated segmentation method can be used with larger VOIs however the VOIs for this study were selected to ensure analysis of only fracture callus and not callus formation around the external fixator pins.

### 2.3 Automated method

Using the selected VOIs, images were subjected to numerous 2D processing steps to achieve callus: cortex separation; this involved implementation of the preprogrammed image processing plugins within CTAn custom processing menu. The first step in this segmentation involves application of appropriate global thresholding (range 150–255 gray scale values) to separate “space” from bone, which simultaneously also removes the majority of the callus due to its lower mineral density. After thresholding, a 1‐pixel radius round kernel “erosion” step that removes remnant callus is followed by a 3‐pixel radius round kernel closing step, which replaces pixels lost within the cortex in the previous step. Ensuing rounds of despeckling to remove remnant callus are followed by morphological operations that replace pixels lost from the cortex during preceding erosion and despeckling. The image is then “despeckled“ to remove connected white pixels (mineralized tissue) (area < 250 pixels) followed by a 2‐pixel radius round kernel closing step. Further “remnant“ callus is removed with a 1‐pixel radius round kernel erosion step and despeckling to remove connected white pixels (mineralized tissue) (area < 250 pixels); pixels lost from cortex are replaced with sequential closing steps consisting of 2 pixels, then a 10‐pixel radius round kernel. Processing proceeds with a 1‐pixel radius round kernel erosion step and two dilation steps of 4 and 2 pixels, respectively. A pair of final despeckling steps to remove connected white pixels (mineralized tissue) (area < 1000 and <100 pixels) effectively removes all remaining callus to retain only cortex. This set of processed images is copied to the clipboard and the original unprocessed image data set reloaded; the reloaded image undergoes a final global thresholding (120–255 gray scale values) step, thus selecting callus and cortex, after which segmented cortex in the clipboard is subtracted from the reloaded data set to leave only callus for analysis in CTAn (Figure 2B).

Due to different scanning systems (same model different machine) being used rat femora required the use of variable global thresholding to improve the segmentation of callus from cortical bone: (threshold values used: 110–255/60–255 [five samples] and 100–255/60–255 [one sample]). The full list of CTAn plugins used can be found in Supporting Information: Figure S1.

### 2.4 Manual segmentation

Using the selected VOIs, callus was manually segmented using CTAn (Bruker), first by segmenting the cortices using the manual ROI drawing tool to select the boundaries of the cortical bone and then using a tool called subtractive ROI to select everything outside of the manually drawn cortical ROI. This callus VOI and the cortical bone segmentations were then analyzed using 2D and 3D analyses within the custom processing tools in CTAn (Bruker).

### 2.5 Validation

Validation of our method was achieved by comparing measurements of callus microarchitecture (tissue volume [TV], BV, and BV/TV) from the automated method with the “gold standard” manually segmented callus VOIs from scans of fractured femora. Nonfractured femora were also subjected to the same methodology in the absence of callus to demonstrate the accuracy in callus identification and separate segmentation from cortices.

### 2.6 Statistical analysis

Statistical analysis was performed using GraphPad Prism. Normality and homogeneity of variance of all data sets were calculated using the Shapiro–Wilk test. Two sample *t*‐test with Welch's correction was used to compare 3D volumetric analyses of auto‐segmented callus (TV, BV, and BV/TV, bone surface [BS], trabecular thickness, and connectivity) between fractured and unfractured femora and ratio paired *t*‐test was used to compare manual segmentation results (for callus: TV, BV, BV/TV, trabecular thickness, and connectivity; for cortical bone: TV, BV, BV/TV, cortical thickness, and mean polar moment of inertia) between users except where normality was not met and then a Wilcoxon matched pairs sign test was used. Pearson's correlation, root mean squared error (RMSE), and Bland–Altman analysis were used to validate the automatic segmentation method against manual segmentation of callus (TV, BV, BV/TV).

2D analytical profile graphs and *p*‐value heatmaps were produced and statistical analysis was performed using RStudio version 1.4.1717 (RStudio: Integrated Development for R. RStudio, PBC). Ratio paired *t*‐test was used to compare 2D analytical results from manual and automatic segmentation methods in both callus and cortical bone from fractured femora. Two‐sample *t*‐test was used to compare 2D analytical results of auto‐segmented callus between fractured and unfractured femora (for callus: tissue area [T.Ar], bone area [B.Ar], tissue perimeter [T.Pm], and bone perimeter [B.Pm]; for cortical: B.Ar, eccentricity, and mean polar moment of inertia).

## 3 RESULTS

### 3.1 Method for automatic segmentation of callus

**Figure 3:**
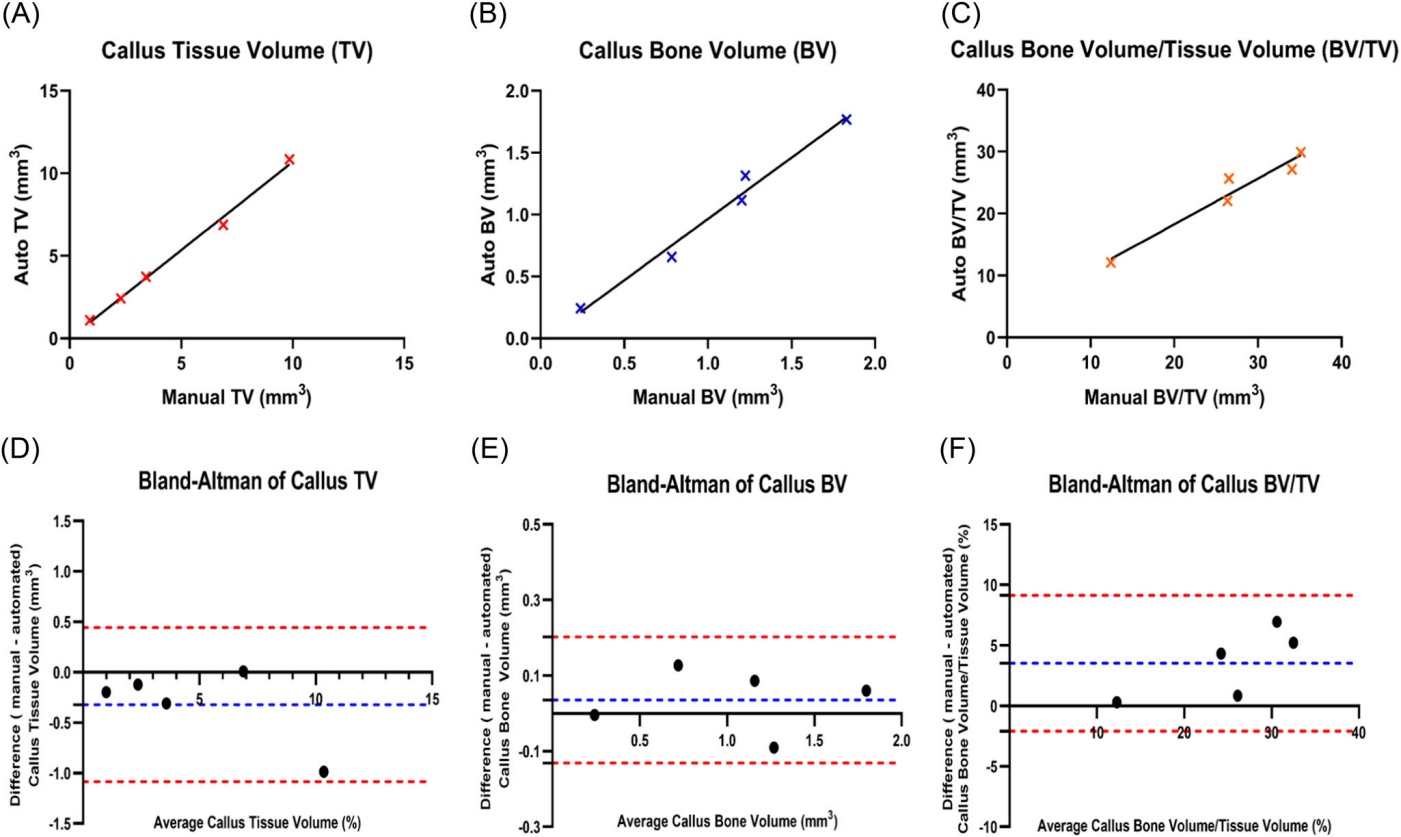
Scatterplots and Bland–Altman plots depicting the agreement between the different quantitative methods of automatic and manual segmentation of mouse fracture callus. Scatterplots with linear regression lines depicting the correlation of 3D volumetric callus results between the manual and automated segmentation methods. This was performed for fracture callus (A) tissue volume (TV), (B) bone volume (BV), and (C) bone volume/tissue volume (BV/TV). Bland–Altman plots showing the difference between paired measurements of manual and automated segmentations for each parameter plotted against the mean of the two measurements. This was performed for (D) fracture callus TV, (E) BV, and for (F) BV/TV. The 95% limits of agreement (−1.96 *SD* and +1.96 *SD*) are indicated by the red dashed lines and the bias is indicated by the blue dashed line. [Color figure can be viewed at wileyonlinelibrary.com]

To test the validity of our automated segmentation method we compared 3D analytical results from manual and automated segmented callus VOIs. Pearson's correlation analysis revealed a strong proportional correlation between measurements from both methods consistent with appropriate segmentation of callus using the automated method. Significant correlation coefficients for TV (*r* = 0.997, RMSE = 0.2962, *p* = 0.0002), BV (*r* = 0.990, RMSE = 0.0848, *p* = 0.0013), and BV/TV (*r* = 0.972, RMSE = 1.616, *p* = 0.0055) were observed when comparing automated and manual segmentation methods, indicating a strong linear relationship (Table 1) (Figure 3A–C). To further test the alliance between the methods we performed Bland–Altman analyses in which the difference between manual and automated measurements were plotted against the averages (Figure 3D–F). This revealed good agreement between methods with mostly low levels of bias: TV bias: −0.3205 mm^3^, BV bias: 0.03583 mm^3^, and BV/TV bias: −3.524%.

**Table 1:** Correlation of results from manual and automatic segmentation of mouse fracture callus

Tissue parameter	Mean ± SD		Pearson's correlation coefficient (*r*)	RMSE
	Manual	Auto		
Callus tissue volume (mm^3^)	4.67 ± 3.65	4.99 ± 3.91	0.9971	0.2962
Callus bone volume (mm^3^)	1.05 ± 0.59	1.02 ± 0.59	0.9896	0.0848
Callus bone volume/tissue volume (%)	26.91 ± 9.07	23.38 ± 6.90	0.9722	1.616

*Note*: Mean and SD, Pearson correlation coefficients (*r*) of measurements from manually and automatically segmented fracture callus of the femur. The mean and SD of tissue volume, bone volume, and bone volume/tissue volume are reported as well as the Pearson's correlation coefficient (*r*) and RMSE obtained from parametric analysis.

Abbreviations: RMSE, root mean squared error; SD, standard deviation.

### 3.2 Manual segmentation is time‐consuming and subject to user variability

Most investigations of fracture repair in experimental models have used manual segmentation and we, therefore, explored the variability and time taken to complete these analyses, to provide comparator data for the automated methodology. Fractured femora (right; *n* = 5) were scanned, followed by manual segmentation of fracture callus from the cortical bone by two independent users with comparable training and experience. Manual segmentation for each bone required approximately 30 min to complete before analysis, whereas the automated method has analysis integrated into the workflow and therefore does not require the additional analysis step that manual segmentations require. Qualitative evaluation of the efficiency of manual segmentation was initially assessed based upon visualization of the fully segmented cortical (white) and callus (red) bone compartments (Figure 2C,D). Analytical results of callus and cortical bone segmentations from two different users were then compared.

Our data show that data sets segmented by different users only differed in measurement of cortical bone thickness. The interuser variation associated with manual segmentation between two different users in all parameters ranged from 1.72% for BV to 4.2% for connectivity (Table 2).

**Table 2:** Interuser variation associated with manual segmentation

	User 1	User 2	*p*‐value Users 1 versus 2	CV (%)
*3D callus measurements*				
Tissue volume (mm^3^)	4.44 ± 3.72	4.45 ± 3.68	0.74	3.66
Bone volume (mm^3^)	1.52 ± 0.88	1.55 ± 0.91	0.53	1.72
Bone volume/tissue volume (%)	41.58 ± 12.56	42.45 ± 12.53	0.38	2.92
Trabecular thickness (mm)	0.058 ± 0.010	0.059 ± 0.012	0.41	1.86
Connectivity	7575 ± 7751	7657 ± 7659	0.78	4.19
*3D cortical measurements*				
Tissue volume (mm^3^)	2.57 ± 0.25	2.56 ± 0.21	0.89	3.20
Bone volume (mm^3^)	1.05 ± 0.12	1.02 ± 0.09	0.35	3.43
Bone volume/tissue volume (%)	40.69 ± 3.92	39.89 ± 3.19	0.35	2.87
Cortical thickness (mm)	0.149 ± 0.013	0.147 ± 0.014	0.02	1.62
Mean polar moment of inertia (mm^4^)	0.82 ± 0.09	0.82 ± 0.07	0.79	2.79

*Note*: Ratio paired *t*‐test was used to compare results of manual segmentation between Users 1 and 2. Data (means ± SD) with statistical significance as *p* ≤ 0.05 and CV (%) between users.

Abbreviation: CV, coefficient of variation.

### 3.3 Nonbiased automatic segmentation is time efficient without user influence

To overcome inefficiencies related to manual segmentation a series of noise reductions, morphological, bitwise, and arithmetical operations were developed (Figure 2B). Implementation of these methods shows that automatic segmentations of the same datasets used for the manual segmentation took ~1 min per data set, a 30‐fold reduction in time. Moreover, implementation of this automated methodology appears to restrict user variation.

Quantitative comparison of auto‐segmented fractured and control/nonfractured femora revealed that callus can be effectively segmented from cortical bone with proper and accurate selection. 3D volumetric analysis of the auto‐segmented callus showed statistically significant differences between fractured and control (nonfractured) femora in all architectural parameters excluding BV/TV, with callus from fractured femora exhibiting higher BV, TV, BS, and connectivity (Table 3).

**Table 3:** Comparison of auto‐segmented callus results between groups

			*p*‐value	Significance
	Control	Fracture	Control versus fracture	*p* ≤ 0.05
*3D callus measurements*				
Tissue volume (mm^3^)	0.02 ± 0.02	4.99 ± 3.91	0.0468	*
Bone volume (mm^3^)	0.004 ± 0.005	1.020 ± 0.590	0.0184	*
Bone volume/tissue volume (%)	24.24 ± 17.38	23.38 ± 6.90	0.5476	NS
Bone surface (mm)	0.96 ± 0.63	122.30 ± 71.94	0.0196	*
Trabecular thickness (mm)	0.02 ± 0.007	0.03 ± 0.003	0.0159	*
Connectivity	37 ± 23.72	19,771 ± 12,725	0.0256	*

*Note*: *t*‐test with Welch's correction was used to compare auto‐segmented callus results from control (nonfractured) versus fracture groups. When normality was not met, a Mann–Whitney test was used. Data represent means ± SD with statistical significance as *p* ≤ 0.05.

**Figure 4:**
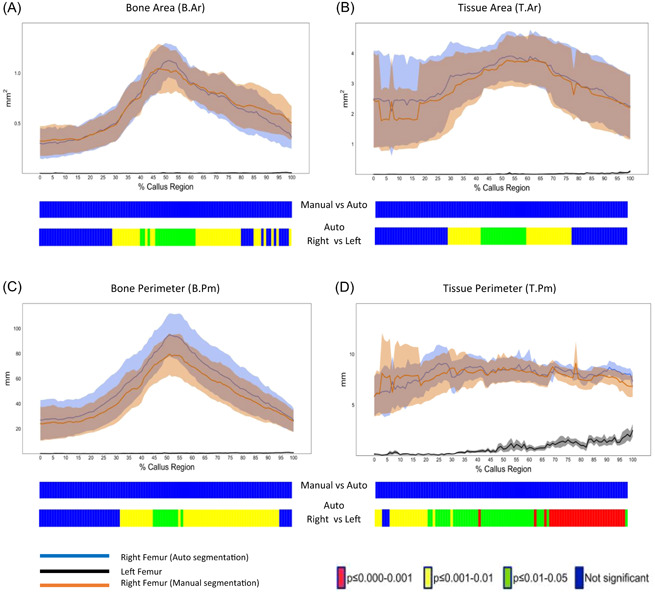
Automatic segmentation can accurately segment callus in fractured femora. micro‐CT 2D morphometric analysis of callus bone of the callus regions in control and fractured femora, from 0% (proximal end) to 100% (distal end), show differences between the automatically segmented fracture group (blue line, *n* = 5) when compared with the control group (black line, *n* = 5) and manually segmented fracture group (orange line, *n* = 5) in: (A) bone area, (B) tissue area, (C) bone perimeter, and (D) tissue perimeter. Line graphs represent the means ± SEM. Two‐sample *t*‐test was used to compare means at each percentile of callus region length between fracture and control groups. The graphical heatmaps (below each graph) display statistical differences at specific matched locations along the length of the callus. Red: *p* ≤ 0.001, yellow: 0.001 < *p* ≤ 0.01, green: 0.01 < *p* ≤ 0.05, and blue: *p* > 0.05. [Color figure can be viewed at wileyonlinelibrary.com]

The efficiency and accuracy of automatic segmentation of callus was further demonstrated by 2D morphometric analysis of the bone architecture along the length of the segmented VOI. Significant differences between fracture and control femora were evident by the 2D analytical profile of the auto‐segmented callus with regard to a number of architectural parameters including T.Ar, B.Ar, B.Pm, and T.Pm. In addition, no significant differences were found between manual and automatic segmentation methods in any parameter or location along the length of the callus region (Figure 4A–D).

**Figure 5:**
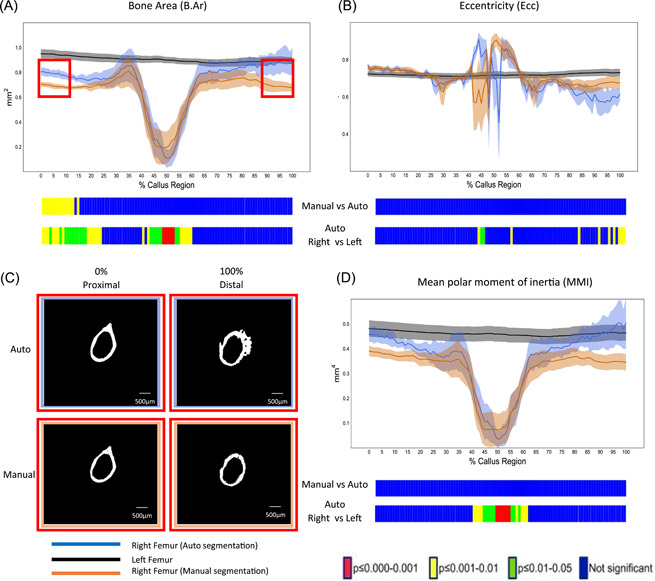
Automatic segmentation can accurately segment cortical bone in fractured and unfractured femora. micro‐CT 2D morphometric analysis of cortical bone of the callus regions of control and fractured of femora from 0% (proximal end) to 100% (distal end) show differences between the automatically segmented fracture group (blue line, *n* = 5) when compared to the control group (black line, *n* = 5), and the manually segmented fracture group (orange line, *n* = 5) in: (A) bone area, (B) eccentricity and (D) mean polar moment of inertia. (C) 2D slice of segmented cortical bone in regions where there is variation between automatic and manual methods. Line graphs (in A, B, and D) represent the means ± SEM. Two‐sample *t*‐test was used to compare means at each percentile of callus region length between fracture and control groups. The graphical heatmaps (below each graph) displays statistical differences at specific matched locations along the length of the callus. Red: *p* ≤ 0.001, yellow: 0.001 < *p* ≤ 0.01, green: 0.01 < *p* ≤ 0.05, and blue: *p* > 0.05. [Color figure can be viewed at wileyonlinelibrary.com]

Comparison of the 2D analytical profiles of the auto‐segmented cortical bone showed clear differences along the segmented regions of fractured and nonfractured femora. At the site of fracture, all the parameters for the fractured femora were less than the nonfractured, excluding eccentricity, revealing a reduced cortical space consisting of less bone, therefore, compromising the predicted strength of the bone which is reflected in the decreased resistance to torsion (Figure 5A–D).

Comparison of 2D analytical profiles between automatic and manually segmented cortical datasets revealed a significant difference in B.Ar in the proximal callus region, this trend was also seen in the distal region but was not significant. However, there is no effect on shape, reflected by eccentricity in these areas as well as having no significant impact on mean polar moment of inertia and therefore predicted strength of the bone (Figure 5A–D).

### 3.4 Automatic segmentation of fracture callus from rat femora

**Figure 6:**
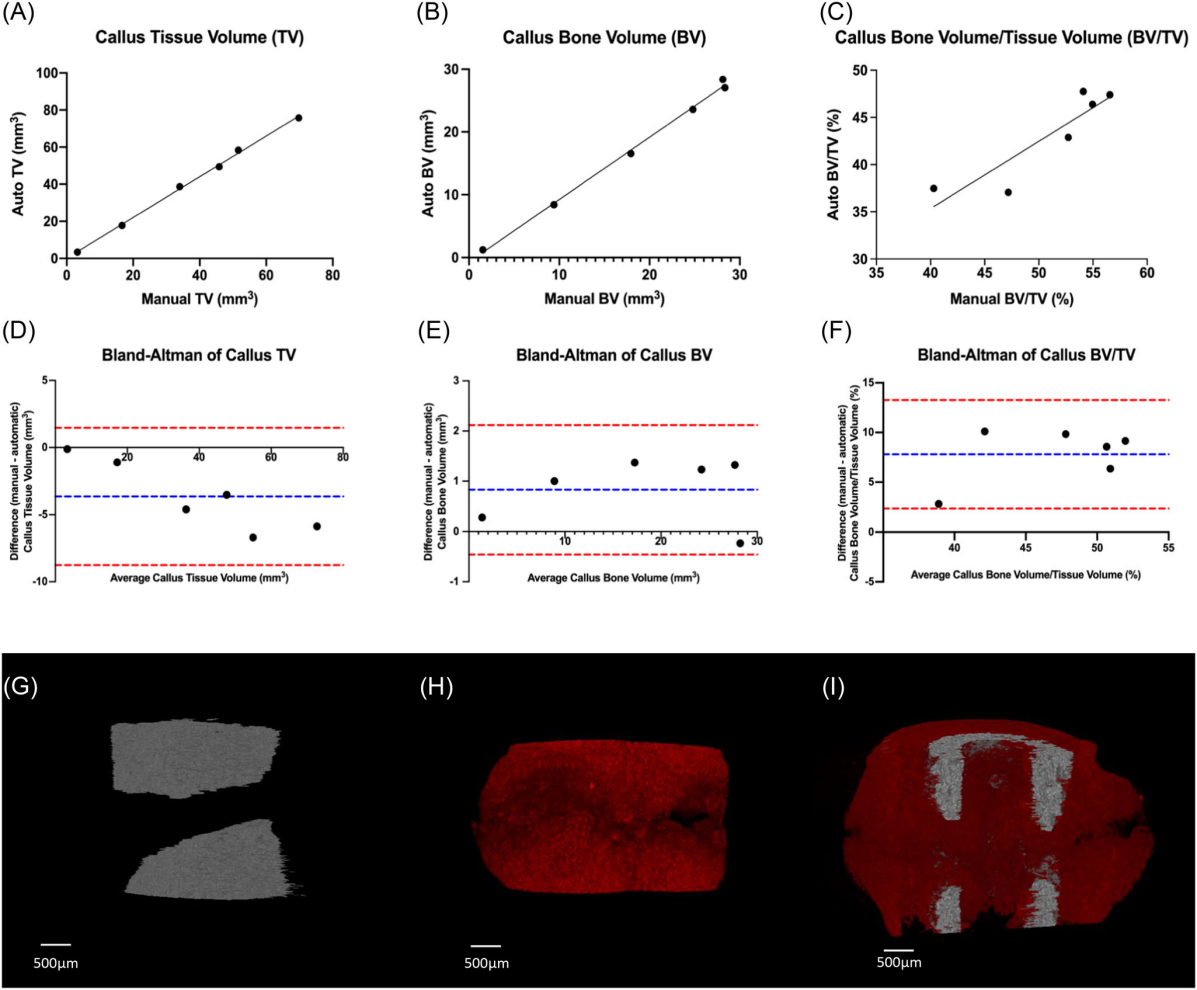
Scatterplots and Bland–Altman plots depicting the agreement between the different quantitative methods of automatic and manual segmentation of rat fracture callus. Scatterplots with linear regression lines depicting the correlation of 3D volumetric callus results between the manual and automated segmentation methods. This was performed for fracture callus (A) tissue volume (TV), (B) bone volume (BV), and (C) bone volume/tissue volume (BV/TV). Bland–Altman plots showing the difference between paired measurements of manual and automated segmentations for each parameter plotted against the mean of the two measurements. This was performed for fracture callus (D) TV, (E) BV, and (F) BV/TV. The 95% limits of agreement (−1.96 *SD* and +1.96 *SD*) are indicated by the red dashed lines and the bias is indicated by the blue dashed line. Volume rendering of automatic segmentation of (G) cortical bone in gray and (H) callus in red and (I) coronal cross‐section. [Color figure can be viewed at wileyonlinelibrary.com]

To test the translatability of our automated segmentation method within rodent species we compared 3D analytical results from manual and automated segmented callus VOIs from rat femora. Using Pearson's correlation analysis, a strong, positive correlation between measurements between manual and automatic segmentation was found, consistent with appropriate segmentation of callus from cortical bone. Significant correlation coefficients for TV (*r* = 0.999, RMSE = 1.138, *p* < 0.0001), BV (*r* = 0.992, RMSE = 0.6510, *p* < 0.0001), and BV/TV (*r* = 0.897, RMSE = 2.155, *p* = 0.0154) were observed, indicating a strong linear relationship (Table 4 and Figure 6A–C).

**Table 4:** Correlation of results from manual and automatic segmentation of rat fracture callus mean and SD and Pearson's correlation coefficients of the measurements in manually and automatically segmented fracture callus

	Mean ± SD		Pearson's correlation coefficient (*r*)	RMSE	BA bias
	Manual	Automated			
Callus tissue volume (TV, mm^3^)	36.871 ± 24.217	40.525 ± 26.590	0.9991	1.138	−3.654
Callus bone volume (mm^3^)	18.365 ± 10.962	17.535 ± 10.896	0.9982	0.6510	0.830
Callus bone volume/TV (%)	50.962 ± 6.136	43.153 ± 4.874	0.8970	2.155	7.809

*Note*: The mean and SD of tissue volume, bone volume, and bone volume/tissue volume are reported. In addition, Pearson's correlation coefficient (*r*) and RMSE were obtained from parametric analysis and BA bias calculated.

Abbreviations: BA, Bland–Altman; RMSE, root mean squared error; SD, standard deviation.

To further test the alliance between these methods we performed Bland–Altman analyses in which the difference between manual and automated measurements were plotted against the averages (Figure 6D–F). This revealed good agreement between methodologies with low levels of bias: TV bias: −3.654 mm^3^, BV bias: 0.830 mm^3^, and BV/TV bias: 7.809%. Qualitative evaluation of the efficiency of this manual segmentation process was initially assessed, based upon visualization of the fully segmented cortical (gray) and callus (red) bone compartments (Figure 6G–I).

## 4 DISCUSSION

Micro‐Computed Tomography (micro‐CT) is a powerful research tool in noninvasive investigation of fracture healing. Architectural parameters of fracture callus are important indices of predicted mechanical strength^12^ and give insights into the fracture healing process. To date, the analysis of fracture callus using micro‐CT has relied upon subjective methods based on user‐defined ROIs and global thresholding only in the segmentation of callus. Herein, we have developed a less subjective, rapid, and comprehensive method for automatic segmentation of callus from cortical bone. This system avoids the introduction of interuser variation and does not rely solely on differing tissue densities/radio opacities to achieve separation.

The method described here is automatic and easily employed with segmentation and analysis taking ~1 min when compared with a manual segmentation that takes ~30 min. Previous methods of callus segmentation from micro‐CT images^2^, ^8^, ^12^ all relied upon global thresholding to separate callus and cortical bone or were limited to segmenting only periosteal callus^2^ and omitting the endosteal callus which is known to contribute to biomechanical stability in femoral fractures.^17^ Reliance upon global thresholding as the basis of a callus segmentation method is restricted due to the increased mineralization of the fracture callus which takes place during healing, making it difficult to define callus and cortical based on greyscale value/mineral density. Our automatic segmentation method, however, does not rely solely on thresholding but uses image processing steps to sever the connections of the callus to the cortical bone independent of the levels of mineral density.

Using micro‐CT tomograms of fractured and unfractured mouse femora, we found that our automatic segmentation method can accurately segment callus from cortical bone to reveal clear differences in bone architectural parameters. These differences are best visualized as 2D morphometric profiles of the callus along the femur. Calculation of Pearson's correlation coefficient showed a strong linear relationship between the manual and automatic segmentations of callus for TV, BV, and BV/TV parameters. Further analysis using Bland–Altman plots determined a good agreement between methodologies with relatively small biases reported for the aforementioned parameters. Comparison of 2D analyses from automatic and manual segmentations along the callus length also demonstrated good agreement with no significant differences found in any parameter. It must be noted that there is larger variation observed in T.Ar for the automatic compared with manual segmentations in regions further away from the fracture site where the transition of callus to cortical bone is more advanced, making distinction increasingly difficult however these differences did not reach significant levels.

Analysis of manually segmented datasets found relatively small interuser variation of 1.7%, 3.7%, and 4.19% for BV, TV, and connectivity, respectively, there is no variation in the same datasets subjected to the automatic segmentation.

The contribution of cortical bone to the callus environment is often overlooked, however, we also demonstrated that this automatic method effectively segments cortical bone as a data set allowing for analysis. Cortical parameters showed lower variation between users compared with callus, however, there was a significant difference in cortical thickness. This was attributed to users finding it difficult to define the cortex and callus as separate ROIs, even within well‐specified sample regions, or due to difficulties to visually identifying their specific boundaries (Figure 2G). This highlights the subjective nature of manual segmentation and the variation introduced by users that is alleviated completely by our automatic segmentation method. However, significant differences were observed in the 2D analytical profiles of cortical bone in the locations most distant from the fracture site, suggesting that automated segmentation of cortical bone becomes inaccurate presumably due to less distinction between cortical and callus bone in these most distant regions (see Figure 5C).

The past two decades have seen an increase in the use of rodent models to study fracture healing, which historically had been assessed in large animals like sheep,^18^ dogs,^19^ and pigs.^20^ Rodent models are particularly useful to study fracture healing and the efficacy of treatment due to the ease and speed of breeding as well as the availability of genetically modified strains and molecular diagnostic tools.^21^ Therefore, we explored the scalability and translatability of our automated segmentation method with the analysis of rat femoral calluses. We show that we can examine the fracture gap in its entirety and successfully segment the callus, with minor changes to the global threshold levels. Several factors, including scanning protocols, equipment, and reconstruction parameters, can affect the greyscale values used for thresholding, although visual assessment and alteration accordingly easily overcome this issue. Correlation analysis revealed a strong linear relationship between manually‐segmented and automatically‐segmented rat fracture callus, with further validation using Bland–Altman plots indicating good agreement with low bias in all architectural parameters. As with mouse fracture callus, the most marked divergence was in BV/TV, likely due to small differences in BV and TV parameters that become amplified or possibly due to difficulties in defining fracture callus where this transitions to cortex.

Beyond these difficulties in efficient segmentation of particular callus areas, we must also consider the fracture model employed in our studies was not the more commonly used Einhorn model^22^ but the model developed by Zwingenberger et al.^13^ The former may allow more bone displacement or fragmentation and further validation of our automated method should therefore be considered before application in other rodent fracture models.

It is noteworthy that our studies segmented the callus at 5 weeks for rats and 6 weeks for mice, which are both later postfracture timepoints than those conducted using other automated methods previously (at 3 weeks).^12^ This later timepoint of healing provides insight into fracture callus architecture beyond the timeline of current automated methods.

Other methods including our own^23^ are described for semiautomated bone segmentation but have not been applied to callus. The excellent methods of Buie et al.^24^ and Kohler et al.^25^ solved many issues related to automated segmentation of cortical from trabecular bone. These were designed for specific morphologies and required certain conditions about bone distribution to be met; relating to the diameter (in pixels) of the largest pore connecting the marrow to the exterior; the former also relied on dual thresholding. Our method addresses such limitations with a simple preprocessing step to isolate the region of interest, which enables all further steps to run automatically. It is important to emphasize, nonetheless, the conceptual similarity of these methods; indeed their independent development highlights their usefulness. Our method also protects against artefactual “clipping” of the outer cortical boundary that can arise in earlier methods if spaces outside the region of interest are smaller than the dilation and erosion amounts; the “cortical mask” grows beyond the image boundaries, while our method retains the periosteal boundary of the segmented cortex. This does not negate the need to further validate our approach in future research via direct comparison with another well‐described approach, such as histology in selected regions. Finally, it is also worth noting that neither the methods of Buie et al.^24^ nor Kohler et al.,^25^ have been applied to the analysis of fracture callus.

In conclusion, we have defined a new automated method for segmentation of callus and cortical bone in micro‐CT tomograms which is easy to use, consistent and comprehensive, allowing for bone microarchitecture analysis across entire fractures. It allows without a priori assumption regarding callus mineralization density predictions of mechanical strength to be made. In addition, as use of rodent models has become a standard in fracture studies, the translatability and scalability of our method in both mice and rats add to its potential utility. It is envisioned that future development of invivo micro‐CT machines may make it feasible to similarly assess fracture repair in humans to establish efficacy of new treatment strategies.

## AUTHOR CONTRIBUTIONS

**Mark Hopkinson**: experimental design, experimental work, and manuscript preparation. **Gareth Jones**: experimental work and manuscript preparation. **Lucinda Evans**: experimental work. **Stephanie Gohin**: experimental work. **Ran Magnusdottir**: experimental work. **Philip Salmon**: experimental work. **Chantal Chenu**: experimental design and manuscript preparation. **Richard Meeson**: experimental work and manuscript preparation. **Behzad Javaheri**: experimental design, experimental work, and manuscript preparation. **Andrew A. Pitsillides**: experimental design, manuscript preparation. All authors have read and approved the final submission.

## CONFLICT OF INTEREST

The authors declare no conflict of interest.

## Supporting information

Supporting information.
