# Supplementary material for: A new method for segmentation and analysis of bone callus in rodent fracture models using micro‐CT
Source: J Orthop Res. 2023 Jan 11;41(8):1717–28. doi: 10.1002/jor.25507 (PMC10947128; doi:10.1002/jor.25507)
Supplement: Supplementary file 1 — Supporting information. [file JOR-41-1717-s001.docx]

**Supplementary Material**

**Supplementary Figure 1**

**Specific task-list for separation of callus from cortex using this method in CTAn involves:**

- GT (150-255 grey values): removes callus & reduces remaining callus: cortex connectivity
- MO: Erosion in 2D space of round kernel (radius=1 pixel) to remove any remnant callus
- MO: Closing in 2D space of round kernel (radius=3 pixels) lost from cortex in previous

step

- DS: Removing white speckles (area <250 pixels) in 2D space to remove any remnant callus
- MO: Closing in 2D space of round kernel (radius=2 pixels) lost from cortex in previous step
- MO: Erosion in 2D space of round kernel (radius=1 pixel) to remove any remnant callus
- DS: Removing white speckles (area <250 pixels) in 2D space to remove any remnant callus
- MO: Closing in 2D space of round kernel (radius=2 pixels) lost from cortex in previous step
- MO: Closing in 2D space of round kernel (radius=10 pixels) lost from cortex in previous

step

- MO: Erosion in 2D space of round kernel (radius=1 pixels) to remove any remnant callus
- MO: Dilation in 2D space of round kernel (radius=4 pixels) lost from cortex in previous

step

- MO: Dilation in 2D space of round kernel (radius=2 pixels) lost from cortex in previous

step

- DS: Removing white speckles (area <1000 pixels) in 2D space to remove any remnant callus
- DS: Removing white speckles (area <100 pixels) in 2D space to remove any remnant callus
- Bitwise operations: remaining image of cortex copied to clipboard
- Reload: original dataset is reloaded
- GT (120-255 grey values): selects all bone including callus
- Arithmetic step: remove segmented cortex (to clipboard) from ‘Reload’ leaving only callus
- 2D Analysis, 3D Analysis
- Save bitmaps, save bitmaps

Abbreviations used: GT, global thresholding, MO, morphological operation, DS, despeckle.
